# Radiotherapy-induced anti-tumor immune response and immune-related adverse events in a case of recurrent nasopharyngeal carcinoma undergoing anti-PD-1 immunotherapy

**DOI:** 10.1186/s12885-018-4295-8

**Published:** 2018-04-06

**Authors:** T. Finazzi, T. Rordorf, K. Ikenberg, G. F. Huber, M. Guckenberger, H. I. Garcia Schueler

**Affiliations:** 1 Department of Radiation Oncology, University Hospital Zurich, Rämistrasse 100, 8091 Zurich, Switzerland; 2 Department of Oncology, University Hospital Zurich, Zurich, Switzerland; 3 Department of Pathology, University Hospital Zurich, Zurich, Switzerland; 4 Department of Otorhinolaryngology, University Hospital Zurich, Zurich, Switzerland

**Keywords:** Nasopharyngeal, Radiotherapy, immunotherapy, Immunostimulation, Pembrolizumab

## Abstract

**Background:**

Treatment of recurrent nasopharyngeal carcinoma is a challenging clinical problem. We report the case of a 46 year old male showing excellent response and signs of immunostimulation following re-re-irradiation for recurrent nasopharyngeal carcinoma under systemic treatment with pembrolizumab.

**Case presentation:**

Patient was first diagnosed with locoregionally advanced, non-keratinizing nasopharyngeal carcinoma in 2010. After achieving complete remission following induction chemotherapy and concurrent curative chemoradiation, the patient subsequently developed distant and locoregionally recurrent disease. He received various treatments (neck dissection, radiotherapy to a bony metastasis, palliative chemotherapy, stereotactic re-irradiation of local recurrence) before initiation of anti- PD-1 immunotherapy with pembrolizumab in January of 2016. Following marked local progression 6 months thereafter, we performed re-re-irradiation of the recurrent tumor after careful evaluation and treatment planning. While treatment was well tolerated, the patient subsequently developed marked clinical and radiological signs of immunostimulation with mucosal irritation and swelling of lacrimal and salivary glands as described in the report. Immunotherapy with pembrolizumab was reinitiated, with re- staging showing excellent response with regression of all tumorous lesions. At the time of this report, following near complete recovery of inflammatory symptoms, the patient remains in excellent condition and free from recurrence under treatment with pembrolizumab.

**Conclusions:**

To our knowledge, we report the first observation of a combined effect of immunotherapy and radiotherapy in a patient with recurrent nasopharyngeal carcinoma. Demonstrating distinct signs of immunostimulation as well as excellent tumor response in a heavily pretreated patient progressing under anti-PD-1 immunotherapy, the case adds to the rising paradigm of an immunostimulatory effect of radiotherapy in patients undergoing treatment with immune checkpoint inhibitors.

## Background

Treatment of recurrent nasopharyngeal carcinoma is a challenging clinical problem. We report the case of a 46 year old male showing excellent response and signs of immunostimulation following re-re-irradiation for recurrent nasopharyngeal carcinoma under systemic treatment with pembrolizumab.

## Case presentation

**Fig. 1:**
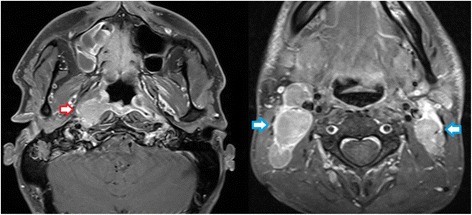
T1-weighted MRI in November of 2010 showing contrast-enhancing right-sided nasopharyngeal primary tumor (red arrow) as well as bilateral metastatic lymph nodes of the neck (blue arrows)

**Fig. 2:**
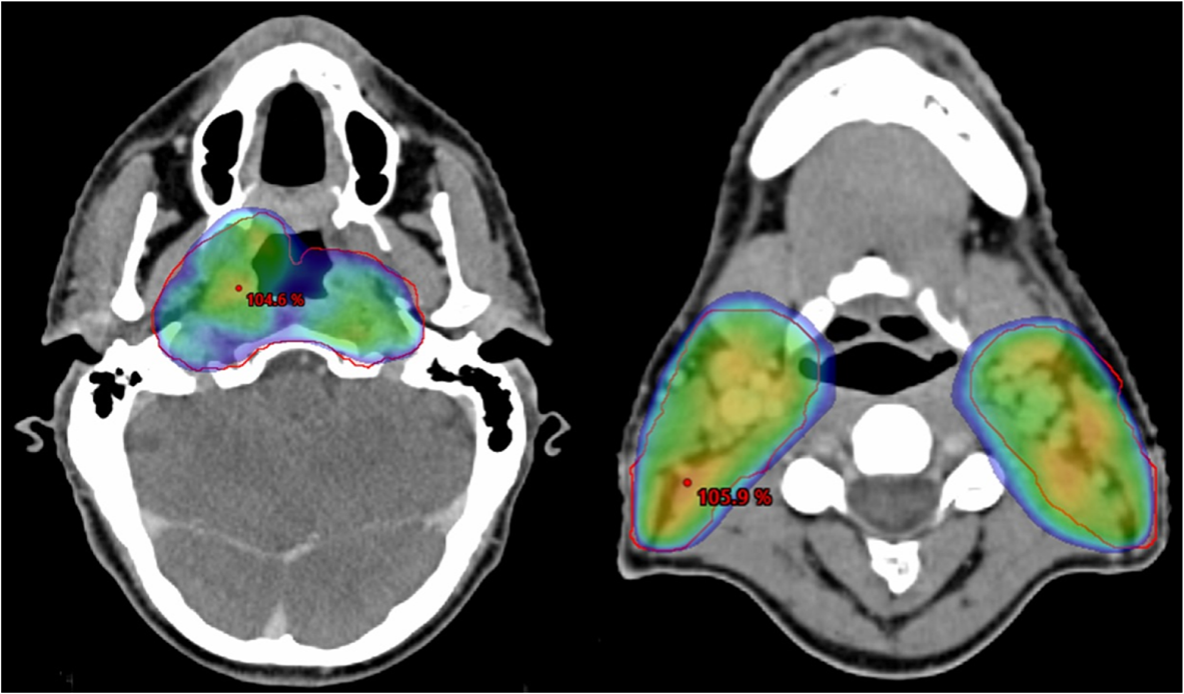
Intensity-modulated radiotherapy plan of 2010 showing 95%-isodose coverage of bilateral cervical lymph nodes with simultaneous integrated boost (SIB) of 70 Gy to the primary tumor and metastatic lymph nodes

Patient was originally diagnosed with locoregionally advanced, non-keratinizing nasopharyngeal carcinoma (stage IVB; cT2 cN3a cM0; EBV-associated) in November of 2010 (Fig. 1). He underwent treatment with neoadjuvant chemotherapy (two 3-weekly cycles of cisplatin 100 mg/m^2^ d1 and fluorouracil 1000 mg/m^2^ d1–4) followed by concurrent curative chemoradiation. Intensity-modulated radiotherapy (volumetric modulated arc therapy; VMAT) was delivered to the primary tumor and nodal metastases in 35 daily fractions of 2 Gy to a total dose of 70 Gy as an integrated boost with elective nodal irradiation to 54 Gy (Fig. 2)*.* Due to cisplatin-induced ototoxicity and anaphylactic reaction to cetuximab, concurrent chemotherapy was delivered with 4 cycles of carboplatin 100 mg/m^2^ weekly (stopped prematurely due to thrombocytopenia). Having achieved complete remission, the patient first presented with distant and locoregionally recurrent disease in October 2011, with PET-CT showing a highly suspicious FDG-avid lesion in the thoracic spine which was diagnosed as bony metastasis after further examination in contrast-enhanced MRI (additional biopsy was omitted after interdisciplinary discussion since clinical significance was deemed low). The patient received radiotherapy (45 Gy in fractions of 3 Gy) to the solitary bony metastasis as well as bilateral neck dissection, removing a total of three metastatic lymph nodes of the left neck. He was subsequently free from recurrence and without symptoms for two years, allowing him to work full-time.

In September of 2013, PET-CT and MRI first raised suspicion of a small bony metastasis of the right occipital condyle and the adjacent clivus. Since this region bordered the initial primary tumor and had therefore received the full dose of 70 Gy during chemoradiation, re-irradiation was declined, given that the patient was asymptomatic. However, the patient developed progressive diplopia due to right-sided abducens nerve palsy after a few weeks. A biopsy of the occipital condyle was performed, confirming cancer recurrence. Due to surrounding dural thickening and contrast enhancement suspicious of meningeal carcinomatosis, the patient received one cycle of intrathecal methotrexate; however, lumbar puncture did not show malignant cells. The patient subsequently received 4 cycles of combination chemotherapy (carboplatin, fluorouracil, docetaxel) with good radiological and clinical response.

Due to progressive diplopia in September of 2014, an MRI was performed, showing tumorous infiltration of the cavernous sinus affecting the abducens nerve. The patient underwent stereotactic re-irradiation (single fraction of 14 Gy to the cavernous sinus) at an external institution using CyberKnife®, achieving mild improvement of symptoms. Due to allergic reaction to carboplatin, systemic treatment was switched to docetaxel and gemcitabine for a total of 6 cycles. The patient was again free from disease progression for one year, before developing tumor progression at the skull base involving the cavernous sinus, Meckel’s cave and the internal carotid artery on the right side as well the middle cranial fossa including the hypoglossal canal in autumn of 2015.

Following the presentation of promising preliminary data for heavily pretreated patients with nasopharyngeal carcinoma in the KEYNOTE-028 cohort [1], a request for medical insurance coverage of immunotherapy with pembrolizumab was made whilst re-challenge chemotherapy with docetaxel and gemcitabine was administered during the decision process. Having received approval, immunotherapy with pembrolizumab was initiated in January of 2016. At this point, PET-CT did not show any distant metastases and circulating EBV DNA (a biomarker for nasopharyngeal carcinoma) was not measurable.

**Fig. 3:**
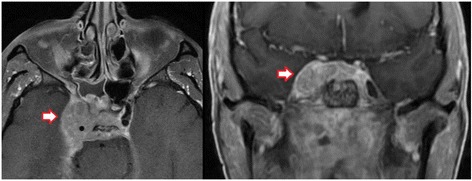
T1-weighted MRI in June of 2016 showing bulky tumor recurrence (red arrows) in axial and coronal plane

**Fig. 4:**
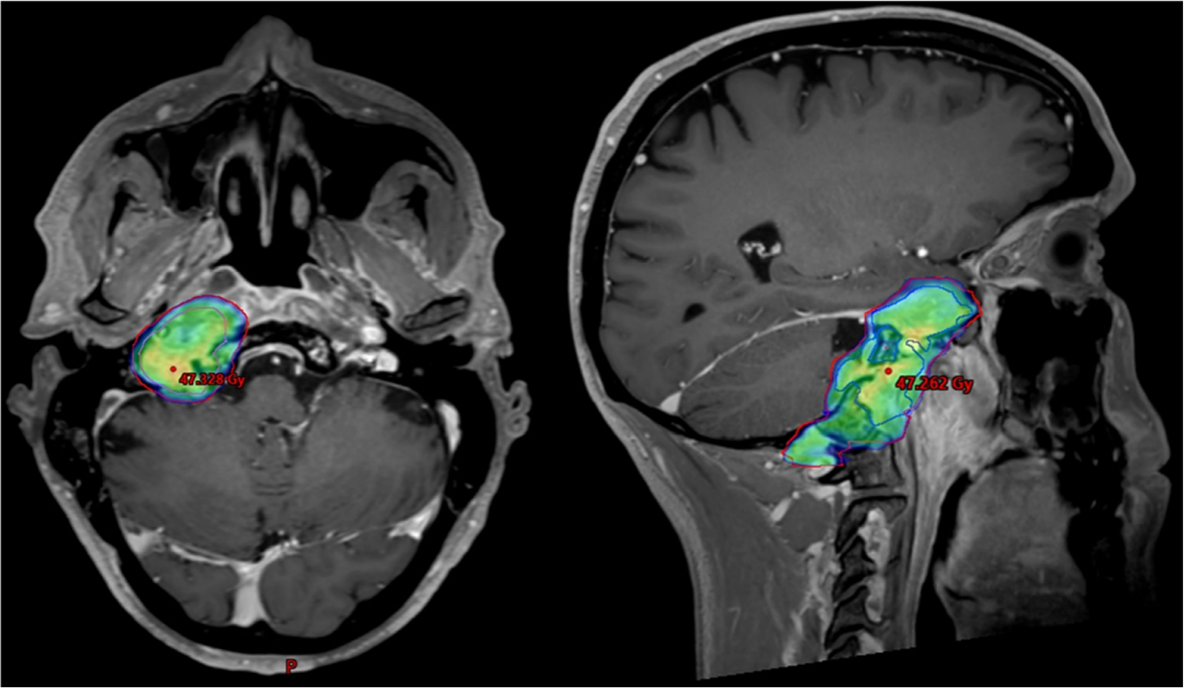
Treatment plan for stereotactic re-re-irradiation using rotational intensity-modulated radiotherapy (volumetric modulated arc therapy; VMAT). The 95%-isodose coverage of the prescribed 45 Gy is shown on fused MRI in axial and sagittal plane, with the latter demonstrating extensive craniocaudal (including prevertebral) tumor extension

While restaging 3 months after initiation of pembrolizumab demonstrated an overall stable situation, the patient progressed again in June of 2016 after 6 months of pembrolizumab, with MRI showing marked increase of tumorous infiltration in the cavernous sinus, Meckel’s cave, the right carotid artery, the occipital condyle as well as the prevertebral space (Fig. 3). During the same period, the patient developed clinical symptoms with progressive trismus, right sided hypoglossal and glossopharyngeal nerve palsy with dysphagia as well as right sided facial hypesthesia, fitting the radiological diagnosis of progressive disease rather than pseudoprogression under immunotherapy. Since the tumor remained unresectable (large tumor extension affecting dura, cavernous sinus, clivus and internal carotid artery without realistic chance of achieving an R0 resection), the patient was once again presented for radiotherapy. After careful evaluation and treatment planning, we performed stereotactic image-guided re-re-irradiation of the recurrent lesion to a total dose of 45 Gy in 25 daily fractions of 1.8 Gy over 6 weeks (Fig. 4). Immunotherapy with pembrolizumab was paused during radiotherapy, which was completed in early October of 2016. Apart from marked fatigue, irradiation was well tolerated, with only mild dysphagia and two episodes of nausea (self-limiting without need for steroids).

Two weeks after treatment completion, the patient first reported symptoms of a common cold with nasal congestion. The patient was seen by our otorhinolaryngologists and received symptomatic treatment and antibiotics (amoxicillin / clavulanic acid) due to thick, putrid mucus in the nasal cavity and the nasopharynx. The patient also reported symptoms of ocular irritation with a burning sensation, dryness and epiphora as well as pronounced swelling of the eyelids in the early morning, also receiving symptomatic treatment after consultation with our ophthalmologists. Having achieved some symptomatic improvement, the patient then presented with a painful swelling of the left submandibular gland suspicious of sialadenitis without sonographic signs of sialolithiasis (fine-needle aspiration was refused by the patient). Days later, he also developed an itching maculopapular rash on the whole body with skin biopsy showing perivascular lymphohistiocytic infiltration mixed with eosinophils. Suspecting cutaneous drug eruption caused by amoxicillin, our dermatologists prescribed topical and systemic steroids which led to slow recovery (over 6 weeks).

**Fig. 5:**
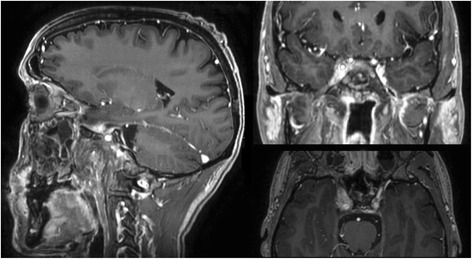
T1-weighted MRI in December of 2016 showing excellent subtotal tumor response following re-re-irradiation in sagittal, coronal and axial plane

**Fig. 6:**
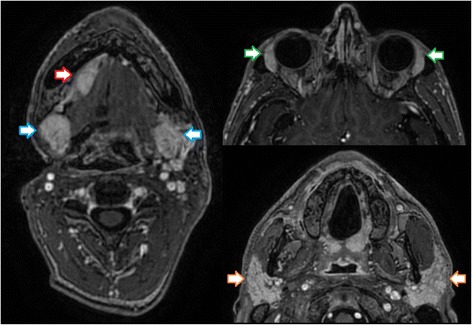
T1-weighted MRI in December of 2016 showing signs of inflammation with marked swelling of salivary glands (sublingual, red arrow; submandibular, blue arrows; parotids, orange arrows) as well as lacrimal glands (green arrows)

**Fig. 7:**
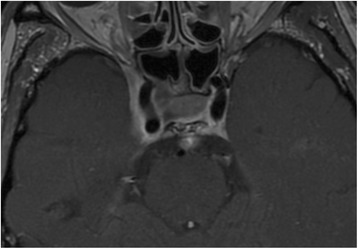
T1-weighted MRI in May of 2017 demonstrating ongoing, subtotal tumor response

Treatment with pembrolizumab was ultimately reinitiated 7 weeks after completion of radiotherapy, and we performed a re-staging with PET-CT and MRI in December of 2016, showing an excellent response with regression of all tumorous lesions (Fig. 5). Notably, a marked swelling of the lacrimal and salivary glands was observed (Fig. 6), which we interpreted as a sign of increased immunologic response to immunotherapy following irradiation. Treatment with pembrolizumab was continued and well tolerated, with slow regression of the inflammatory symptoms described above as well as remarkable neurological improvement (dysphagia, diplopia, trigeminal function). Fittingly, re-staging MRI in February and May of 2017 showed an ongoing response with further regression of the tumorous lesions in MRI (Fig. 7). Following near complete recovery of inflammatory symptoms, PET-MRI in August of 2017 showed regression of inflammatory signs as well as ongoing local control without distant metastases (almost 6 years after first receiving radiotherapy for a bony metastasis). At the time of this report, the patient is in excellent condition, continuing treatment with pembrolizumab as well as rehabilitative treatments under regular follow-up.

## Discussion

Treatment of recurrent nasopharyngeal carcinoma remains a significant clinical problem, with local recurrence representing a major cause of mortality and morbidity [2]. Management of these patients is challenging, with salvage strategies including surgery as well as various forms of radiotherapy (external intensity-modulated radiotherapy (IMRT) including stereotactic radiotherapy or radiosurgery, proton irradiation, brachytherapy) with or without chemotherapy [2–16].

We assume that the case discussed demonstrates distinct signs of a combined effect of immunotherapy and radiotherapy in a heavily pretreated patient with recurrent nasopharyngeal carcinoma progressing under anti-PD-1 immunotherapy. To our knowledge, it is thus the first reported observation of such an effect in a patient with recurrent nasopharyngeal carcinoma. Showing both an excellent local response as well as clinical and radiological inflammatory signs outside of the radiation field, the case adds to the rising paradigm of an immunostimulatory effect of radiotherapy in patients undergoing therapy with immune checkpoint inhibitors [17–19].

While differentiating delayed effects of immunotherapy from responses caused partially or completely by local treatment is intrinsically difficult, the chronological association with radiotherapy as well as the regional distribution of inflammatory effects (not solely accountable to direct radiation effects due to the dose distribution) seem striking. Since the first clinical signs of a possible immunostimulation appeared 10 months after initiation of pembrolizumab and tumor response was first seen 2 months thereafter, the time to response would be on the far end of what has been reported for anti-PD-1 immunotherapy in various types of cancer, with most clinical trials showing a median time to response of around 2 months [20–29]. Notably, re-irradiation using modern external beam radiation techniques to apply high biological doses (with or without brachytherapy) has been shown to achieve adequate local control rates in various series of recurrent nasopharyngeal carcinoma, utilizing both standard fractionation as well as hypofractionated regimens and sometimes chemotherapy as a radiosensitizer [30–32]. It is therefore difficult to discriminate good local control achieved by radiation from synergistic effects of any bimodality treatment. However, given the extent and duration of response to a very moderate dose of 45 Gy in fractions of 1.8 Gy, we considered attribution of local control solely to radiotherapy as very unlikely, particularly since parts of the target volume had previously been re-irradiated using CyberKnife® without lasting effect.

Checkpoint inhibitor immunotherapy is associated with a unique spectrum of immune-related adverse events, reflecting its underlying mechanisms of action aiming at T cell activation and enhanced antitumor immune response [33, 34]. While dermatologic toxicity is the most common (and often the earliest) inflammatory side effect, possible immune-related adverse events include mucosal and gastrointestinal toxicities, pneumonitis, hepatotoxicity, endocrinopathies and (more rarely) neurologic, renal, pancreatic and ocular toxicities [35]. Although the clinical signs seen in our patient (mucosal, including nasal and ocular, irritation; swelling of lacrimal and salivary glands; maculopapular rash) might well represent disorders of infectious and allergic nature, we believe that the assumption of immunostimulation as a common cause following irradiation under anti-PD-1 immunotherapy is reasonable, given the clinical course. Of note, dermatologic toxicities and radiological signs of immune-related adverse events have been associated with better outcome in patients treated with checkpoint inhibitor immunotherapy, although generalization of these observations is not possible [36–39].

The concept of a synergistic effect of radiotherapy and immune checkpoint inhibitors aiming at enhanced response rates and potential long term tumor control is based on an increasing amount of encouraging preclinical and clinical evidence [17–19, 40–44]. Radiation hereby acts as an immune stimulus, recruiting cytokines that enable anti-tumor responses within and outside the radiation field [45]. While the immunogenic properties of radiation (including observations of an abscopal effect following radiotherapy as an otherwise rare clinical phenomenon) have long been discussed, the emergence of immunotherapy and the growing amount of preclinical data have moved the rationale of a combined treatment approach into the spotlight of research, potentially indicating a paradigm shift in the utilization of radiotherapy in these patients [45–47]. Understandably, these observations have also sparked a particular interest in the management of heavily pretreated patients with limited options for effective salvage treatment, such as the case presented. However, with trials combining radiotherapy and immune checkpoint inhibitors currently ongoing [45], the remaining uncertainties surrounding the role and clinical implication of these approaches need to be emphasized.

In summary, we observed an excellent response to combined immunotherapy and re-re-irradiation in a case of recurrent nasopharyngeal carcinoma, and we are hopeful to see future developments that improve outcomes for these patients with otherwise limited treatment options.

## Abbreviations

DNA: Deoxyribonucleic acid
EBV: Epstein-Barr Virus
FDG: Fluorine-18 (F-18) fluorodeoxyglucose
Gy: Gray
IMRT: Intensity-modulated radiotherapy
MRI: Magnetic Resonance Imaging
PD-1: Programmed cell death protein 1
PET-CT: Positron emission tomography–computed tomography
PET-MRI: Positron emission tomography-magnetic resonance imaging
SIB: Simultaneous integrated boost
VMAT: Volumetric modulated arc therapy
